# Combination of pseudoephedrine and emodin ameliorates LPS-induced acute lung injury by regulating macrophage M1/M2 polarization through the VIP/cAMP/PKA pathway

**DOI:** 10.1186/s13020-021-00562-8

**Published:** 2022-02-05

**Authors:** Wen-Ba Wang, Jing-Tao Li, Yi Hui, Jie Shi, Xu-Yan Wang, Shu-Guang Yan

**Affiliations:** 1 College of Basic Medicine, The Shaanxi University of Chinese Medicine, Xianyang, 712046 China; 2 Departments of Infectious Disease, The Affiliated Hospital of Shaanxi University of Chinese Medicine, Xianyang, China; 3 Departments of Respiratory Diseases, The Affiliated Hospital of Shaanxi University of Chinese Medicine, Xianyang, China; 4 Department of Endocrinology, Genetics and Metabolism, The Rainbow Hospital of Xianyang, Xianyang, China

**Keywords:** Acute lung injury, LPS, Pseudoephedrine + emodin, Macrophages, VIP

## Abstract

**Background:**

Acute lung injury (ALI) is an acute multifactorial infectious disease induced by trauma, pneumonia, shock, and sepsis. This study aimed to investigate the protective effects of pseudoephedrine and emodin combined treatment in experimental ALI, as well as the mechanisms underlying the regulation of inflammation and pulmonary edema via the VIP/cAMP/PKA pathway.

**Methods:**

The wistar rats were randomly divided into fifteen groups (n = 5). Rats in each group were given intragastric administration 1 h before LPS injection. Those in the control and LPS groups were given intragastric administrations of physiological saline, rats in other groups were given intragastrically administered of differential dose therapeutic agents. The rats in the LPS and treatment groups were then injected intraperitoneally with LPS (7.5 mg/kg) to induce ALI. After being treated with pseudoephedrine and emodin for 12 h, all animals were sacrifice. Anal temperatures were taken on an hourly basis for 8 h after LPS injection. Pathological examination of lung specimen was performed by H&E staining. Cytokines (IL-1β, TNF-α, IL-6, iNOS, IL-10, Arg-1, CD86, CD206, F4/80, VIP) in lung tissue were assayed by ELISA and immunofluorescence. The expression of VIP, CAMP, AQP-1, AQP-5, p-PKA, PKA, p-IκBα, IκBα, p-p65, p65, p-P38, P38, p-ERK1/2, ERK1/2, p-JNK1/2, JNK1/2 protein in lung was determined by western blotting.

**Results:**

After rats being treated with pseudoephedrine + emodin, reduced of fever symptoms. The contents of inflammatory cytokines (IL-1β, TNF-α, IL-6, iNOS) were decreased and anti-inflammatory cytokines (IL-10, Arg-1) were significantly increased in serum. Pseudoephedrine + emodin treatment effectively promoted VIP cAMP and p-PKA protein expression in lung tissues, and significantly inhibited NF-κB, MAPK phosphorylation, Pseudoephedrine + emodin treatment can inhibit M1 polarization and promoted M2 polarization via the VIP/cAMP/PKA signaling pathway.

**Conclusions:**

The combination of Pseudoephedrine and emodin was effective in ameliorating LPS-induced ALI in rats by inducing VIP/cAMP/PKA signaling. Inhibiting the NF-κB, MAPK inflammatory pathway, relief of pulmonary edema suppressing macrophage M1 polarization, and promoting macrophage M2 polarization.

## Background

ALI is an acute multifactorial infectious disease induced by trauma, pneumonia, shock, and sepsis [1, 2]. The mortality rate of ALI is 30–50% [3], and survivors live with significantly compromised quality of life resulting from severe damage to lung functions [4]. ALI is characterized by inflammatory cell infiltration, massive production of inflammatory mediators, and diffuse lung inflammation [5]. It is recognized as one of the most direct causes of death in patients with sepsis, in which the intestinal barrier function is impaired and a large number of bacteria and endotoxins in the intestine invade the circulation through the portal vein and lymphatic system, thereby causing lung and intestinal infections and triggering multi-organ dysfunction syndrome [6, 7].

ALI rats are commonly used as animal models in pharmacological studies of lipopolysaccharide (LPS)-induced ALI. It is widely known that LPS activates the expression of inflammatory cytokines, such as interleukin-6 (IL-6) and tumor necrosis factor-α (TNF-α), and induces NF-kB signaling, ultimately leading to cytokine storm and endotoxic shock [8]. The histological nature of ALIs was initially considered to be diffuse alveolar damage; this has now been supplemented with capillary congestion, pulmonary atelectasis, intra-alveolar hemorrhage, alveolar edema, as well as the resulting proliferation of epithelial cells and interstitial pulmonary edema, with particular emphasis on alveolar and interstitial edema triggered by inflammatory exudation as an important pathological aspect of ALI [9]. Macrophages are central nodes of immune activity that demonstrate high plasticity and versatility in response to changes in the body's internal environment; they can exhibit both pro-inflammatory and anti-inflammatory properties in response to LPS stimulation [10]. Depending on the stage of ALI development, macrophages could be polarized to one of two different phenotypes: classically activated macrophage M1, or alternatively activated macrophage M2. During the early stages of inflammation, M1-mediated NF-κB signaling induces the release of several cytokines, including TNF-α, IL-6, IL-1β, chemokines, and granulocyte colony-stimulating factors (G-CSF) [11]. M1 macrophages can further differentiate into TH1 and TH17 pro-inflammatory cells, which increases inflammatory response, but excessive production of pro-inflammatory factors, reactive oxygen species, and reactive nitrogen species could lead to tissue damage [12]. During the inflammation suppression stage, macrophages are typically polarized into M2 macrophages, which have anti-inflammatory repair functions and are typically induced by classical TH2 cytokines, such as IL-4, IL-10 and glucocorticoids.

Vasoactive intestinal peptide (VIP) is one of the most important immunoreactive neuropeptides in the lung; it has several notable biological effects, such as reducing granulocyte recruitment [13] and promoting the expression of the anti-inflammatory factors IL-4 and IL-10 [14]. VIP agonists have been found to exhibit protective properties when used to treat asthma, pulmonary hypertension, chronic bronchitis and pulmonary fibrosis [15]. Cyclic adenosine monophosphate (cAMP is an important substance for tissue and cell metabolism and the maintenance of physiological functions; it can activated through different means, of which hormones and neurotransmitters are some of the most important. As an immunoneuropeptide, VIP can bind to G protein-coupled receptors to activate adenylate cyclases (ACs) and promote cAMP release [16]. Protein kinase (PKA) is a major cAMP target and cAMP can bind to the regulatory subunit of PKA to induce PKA phosphorylation, thereby forming the VIP/cAMP/PKA signaling pathway [17].

In traditional Chinese Medicine, connections between the physiological functions of the lung and the intestines are often emphasized when treating diseases of the lung and intestine. This treatment approach, which translates to “lung-intestine combinational therapy”, tends to employ herbal preparations made from rhubarb and *Ephedra sinica*. The dried stems and leaves of ephedra are traditionally prescribed as a treatment for cough and asthma [18], the herb exhibits a certain degree of toxicity, but it remains popular in China and Japan as a remedy for symptoms related to upper respiratory tract infections—especially cough and fever [19, 20]. One of main active ingredients of ephedra is pseudoephedrine, which produces notable anti-inflammatory effects [21]. Pseudoephedrine is a sympathomimetic drug that acts directly on alpha-adrenergic receptors. Its molecular formula is C_10_H_15_NO, which helps to treat symptoms of the common cold and flu, sinusitis, asthma, and bronchitis [22, 23]. Studies have also demonstrated that pseudoephedrine, when taken after antibiotics as an adjuvant treatment for symptom relief by patients with acute respiratory infections (ARIs), was effective in improving respiratory symptoms, especially those of nasal congestion and sinus headache [24]. The main active component of rhubarbs is emodin, which is a natural anthraquinone derivative, its molecular formula is C_15_H_10_O_5_. Recent studies have revealed a wide range of pharmacological properties of emodin, including anticancer, hepatoprotective, anti-inflammatory, antioxidant, and antibacterial properties [25]. We conducted preliminary experiments in which ALI rats were treated with pseudoephedrine and emodin through gavage administration and discovered significant suppression of inflammation and pulmonary edema. We hypothesized that this could be related to elevated VIP expression in the serum and bronchoalveolar lavage fluid (BALF) and endeavored to further investigate the protective effects of this pseudoephedrine + emodin combination on rats with LPS-induced ALI, as well as the mechanisms underlying the regulation of inflammation and pulmonary edema via the VIP /cAMP/PKA pathway.

## Methods

### Chemicals and reagents

Pseudoephedrine and emodin (purity > 99%) were purchased from Baoji Chenguang biology Ltd. LPS was purchased from Sigma-Aldrich Co. ELISA kits for IL-1β, TNF-α, IL-6, iNOS, IL-10, Arg-1 and VIP were obtained from Elabscience Biotechnology Co. Ltd. (Wuhan, China). Antibodies against AQP-1, AQP-5 VIP, CAMP, PKA/phospho-PKA, IκBα/phospho-IκBα, NF-κB p65/phospho-NF-κB p65, and β-actin were purchased from Affinity Biosciences Ltd. (OH, USA). HRP-conjugated secondary antibodies were purchased from Wuhan Boster Biological Technology LTD. (Wuhan, China). Trizol and Western blot chemiluminescence reagents were obtained from Ambion Thermo Fisher Scientific-CN (Shanghai, China).

### Animal model construction and sampling

Wistar rats were randomly divided into the following treatment groups: control group (n = 5), LPS group (7.5 mg/kg, n = 5), Pseudoephedrine group (40 mg/kg, n = 5), emodin group (80 mg/kg, n = 5), Pseudoephedrine + emodin group (20 + 40 mg/kg, n = 5), Pseudoephedrine treatment group (10 mg/kg, 20 mg/kg, 40 mg/kg, LPS [7.5 mg/kg], n = 5), emodin treatment group (40 mg/kg, 60 mg/kg, 80 mg/kg, LPS [7.5 mg/kg], n = 5), Pseudoephedrine and emodin combined treatment group (5 + 20 mg/kg, 10 + 40 mg/kg, 20 + 40 mg/kg, LPS [7.5 mg/kg], n = 5), or Dex treatment group (3.5 mg/kg, LPS [7.5 mg/kg], n = 5). Rats in each group were given intragastric administration 1 h before LPS injection. Those in the control and LPS groups were given intragastric administrations of physiological saline, rats in other groups were given intragastrically administered of differential dose therapeutic agents. The rats in the LPS and treatment groups were then injected intraperitoneally with LPS (7.5 mg/kg) to induce ALI. In the control, LPS, and combined treatment groups, the rectal temperature of the rats was measured every hour from 0 to 8 h after LPS injection. After 12 h of LPS injection, the rats were anesthetized with an intraperitoneal injection of 10% chloral hydrate (3 mL/kg). The left ventricular arterial blood was collected and centrifuged at 3000*g* for 10 min. Then, the serum was extracted and stored in the refrigerator at − 80℃. The bronchoalveolar lavage (BALF) was performed. All lungs were lavaged with three instillations of 2 mL of normal saline. The lavages were combined and centrifuge for 10 min at 10,000 rpm to remove the cells; the supernatant was then aspirated and frozen using liquid nitrogen. The D/W (dry/wet) ratio of the lower lobe of the right lung was then evaluated. The middle lobe of the right lung was fixed with 4% paraformaldehyde for pathological staining, immunohistochemistry, and immunofluorescence analysis. The remaining lung tissues were stored at − 80 °C for further analysis.

### Isolation and culture of alveolar macrophages from rat BALF

The wild male Wistar rat weighing 200–250 g was anesthetized with 1.5% sodium pentobarbital (35 mg/kg) and fixed on a rat plate. The neck was disinfected in accordance with routine procedures, and the skin of the neck was incised to fully expose the trachea; an incision was made in the middle of the trachea, followed by surgical intubation and fixing. The lung was washed ten times; during each lavage, 6 ml of saline was injected into the lung cavity, the lung was gently rubbed, then lavage fluid was withdrawn. BALF was collected and filtered through a 200 mesh filter. The filtrate was centrifuged at 1000*g* for 10 min and the supernatant was discarded. The pellet was washed twice by centrifugation with PBS at 1000*g* for 10 min. These cells were seeded in sterile 6-well culture dishes and incubated at 37 °C in an incubator containing 5% CO_2_.

### Histopathological analysis

Lung tissues were fixed with 4% paraformaldehyde; these were embedded in paraffin sections (2 μm thick), de-paraffinized, and then rehydrated with an ethanol gradient. Lung tissues were treated with hematoxylin and eosin (H&E) for staining and photographed under light microscopy. The results were graded on a scale of 0 to 3 points by an experienced pathologist in accordance with established procedure from the literature [26].

### Changes in anal temperature and thermal response index (TRI)

Rat body temperatures were measured on an hourly basis for 8 h after LPS injection; these were calculated as ΔT. Thermal response index (TRI, °C × h) was calculated based on the area between the temperature–time curve and the x-axis [27], specifically, it was calculated as the summation of the area of each trapezoid under every two data points. The area of each such trapezoid from t_i_ to t_i+1_ was calculated as (t_i+1_ − t_i_) × (ΔT_i_ + ΔT_i+1_)/2.

### Lung dry/wet (D/W) weight ratio

Samples from the lower lobe of the right lung were collected 24 h after LPS stimulation, blotted with filter paper to remove excess water and weighed immediately (wet weight), and then dried continuously in an oven at 80 °C for 48 h to obtain the dry weight. Tissue edema was assessed by the ratio of wet lung weight to dry lung weight.

### Enzyme-linked immunosorbent assay for the quantification of cytokines

Levels of IL-1β, TNF-α, IL-6, iNOS, IL-10, Arg-1 and VIP in rat serum, BALF and cultured macrophages were measured by ELISA kits. The optical density of the microtiter plate was read at 450 nm.

### Immunohistochemical analysis

Paraffin sections (3 μm thick) were de-paraffinized and then rehydrated with an ethanol gradient. Sections were incubated with 3% H_2_O_2_ to remove endogenous peroxidase, followed by incubation with anti-F4/80 antibodies (1:100, rabbit) overnight at 4 °C. It was then further incubated with HRP-conjugated secondary antibodies for 30 min at 37 °C, stained with hematoxylin, and photographed under sealed conditions.

### Immunofluorescence analysis

Cell slides were de-paraffinized, rehydrated with an ethanol gradient, and 50% TritonX was added dropwise to the tissue and left for 10 min at room temperature. They were then washed with PBS thrice with 5 min each time. Citrate buffer was added to the slide and antigen retrieval was carried out at high temperature for 5 min. The slides were washed with 0.01 M PBS and closed with goat serum for l0 min at room temperature. Sections were incubated overnight at 4 °C with primary antibodies anti-CD86 (1:50; BIOSS, Beijing, China), anti-IL-6 (1:50; BIOSS, Beijing, China), anti-CD206 (1:50; Abcam, Shanghai, China), anti- IL-10 (1:50; Abcam, Shanghai, China). Unbound primary antibodies were washed off with PBS, and sections were incubated with secondary antibodies followed by nuclear staining and image acquisition. Lung tissue sections were incubated with primary antibodies anti-CD80 (1:50; BOSTER, Wuhan, Hubei, China), anti-IL-1β (1:100; BOSTER, Wuhan, Hubei, China), anti-IL-10 (1:50; BOSTER, Wuhan, Hubei, China) and anti-F4/80 (1:100; BOSTER, Wuhan, Hubei, China). Subsequent steps were the same for cellular immunofluorescence.

### Western blot analysis

Lung tissue samples were lysed in RIPA buffer containing protease and phosphatase inhibitors for 30 min. Protein concentrations were measured using the BCA assay kit (Beyotime, China). Proteins were denatured by heating in a 95 °C water bath. Electrophoresis was carried out to transfer proteins to PVDF membranes. Membranes were incubated overnight at 4 °C with the primary antibodies β-actin, VIP, CAMP, AQP-1, AQP-5, PKA, p-PKA, IκBα, p-IκBα, p65, p-p65,P38, p-P38, ERK1/2, p-ERK1/2, JNK1/2 and p-JNK1/2 then washed thoroughly three times with PBST and incubated with HRP-conjugated secondary antibodies (1:5000 dilution) for 1 h at room temperature. Bands were detected with ECL (AmershamPharmacia Biotech, Piscataway, NJ) and the intensity of the bands was quantified using the Image-J gel analysis software.

### Real-time PCR analysis

Total RNA was extracted from cells using Trizol reagent (Ambion, China). cDNA was reverse transcribed using TransScript First-strand cDNA Synthesis Kit (VAZYME, China) and stored at − 80 °C. Relative gene expression was quantified with QuantStudio 6 (ABI, USA) and 0.4 μg of total RNA was reverse transcribed under the following PCR reaction system: ①Pre-denaturation: 95 °C, 10 min, 1 cycle. ② Denaturation: 95 °C, 15 s, 40 cycles. ③Annealing and extension: 60 °C, 60 s, 40 cycles. The following primers were used: GAPDH, *forward 5′-ATGGGTGTGAACCACGAGA-3′ and reverse 5′-CAGGGATGATGTTCTGGGCA-3′, product size 229 BP;* IL-6, *forward 5′-CACAGAGGATACCACTCCCAACAGA-3′ and reverse 5′-ACAATCAGAATTGCCATTGCACAAC-3′, product size 124 BP;* TNF-α*, forward 5′-AGCACAGAAAGCATGATCCG-3′ and reverse 5′-CTGATGAGAGGGAGGCCATT-3′, product size 212 BP;* iNOS*, forward 5′-TTGGCTCCAGCATGTACCCT-3′ and reverse 5′-TCCTGCCCACTGAGTTCGTC-3′, product size 121 BP;* IL-10*, forward 5′-GCTGGACAACATACTGCTAACCG-3′ and reverse 5′-CACAGGGGAGAAATCGATGACAG-3′, product size 218 BP;* Arginase-1*, forward 5′-ATCGTGTACATTGGCTTGCG-3′ and reverse 5′-CGTCGACATCAAAGCTCAGG-3′, product size 184 BP;* Ym-1*, forward 5′-TGGAATTGGTGCCCCTACAA-3′ and reverse 5′-CCACGGCACCTCCTAAATTG-3′, product size 239 BP*. Lung tissue RNA were extracted using the same steps as the those for the cells, but using the following primers: β-actin, *forward 5′-CACGATGGAGGGGCCGGACTCATC-3′ and reverse 5′-TAAAGACCTCTATGCCAACACAGT-3′, product size 240 BP*; TNF-α, *forward 5′-CCGATTTGCCATTTCATACCAG-3′ and reverse 5′-TCACAGAGCAATGACTCCAAAG-3′, product size 232 BP*; IL-1β, *forward 5′-CAGGTCGTCATCATCCC-3′ and reverse 5′-TCAAATCTCACAGCAGCAT-3′, product size 190 BP*; NLRP3, *forward 5′-CTGCTGAAGTGGATCGAAGTG-3′ and reverse 5′-TGCAAAAGGAAGAAACCACGT-3′, product size 187 BP*; VIP, forward *5′-ATCCAGAAGCAAGCCTCAGT-3′ and reverse 5′-ATAGGGCGTGTCATTCTCCG-3′, product size 211 BP*; IL-10, *forward 5′-CAGTCAGCCAGACCCACAT-3′ and reverse 5′-GGCAACCCAAGTAACCCT-3′, product size 141 BP*; Arg-1, *forward 5′-GGTAGCAGAGACCCAGAAGA-3′ and reverse 5′-CAGCGGAGTGTTGATGTCAG-3′, product size 151 BP*. The relative amount of mRNA was calculated using the comparative Ct (ΔCt) method and compared with GAPDH and β-actin.

### Statistical analysis

All quantitative data were expressed as mean ± SD. Statistical analysis was carried out using SPSS 19.0 (IBM). Comparisons between experimental groups were conducted using one-way ANOVA, whereas multiple comparisons were carried out using the LSD method. Statistical significance was defined as p < 0.05, p < 0.01 or p < 0.001.

## Results

### Pseudoephedrine and emodin treatment alleviated LPS-induced ALI rats

**Fig. 1:**
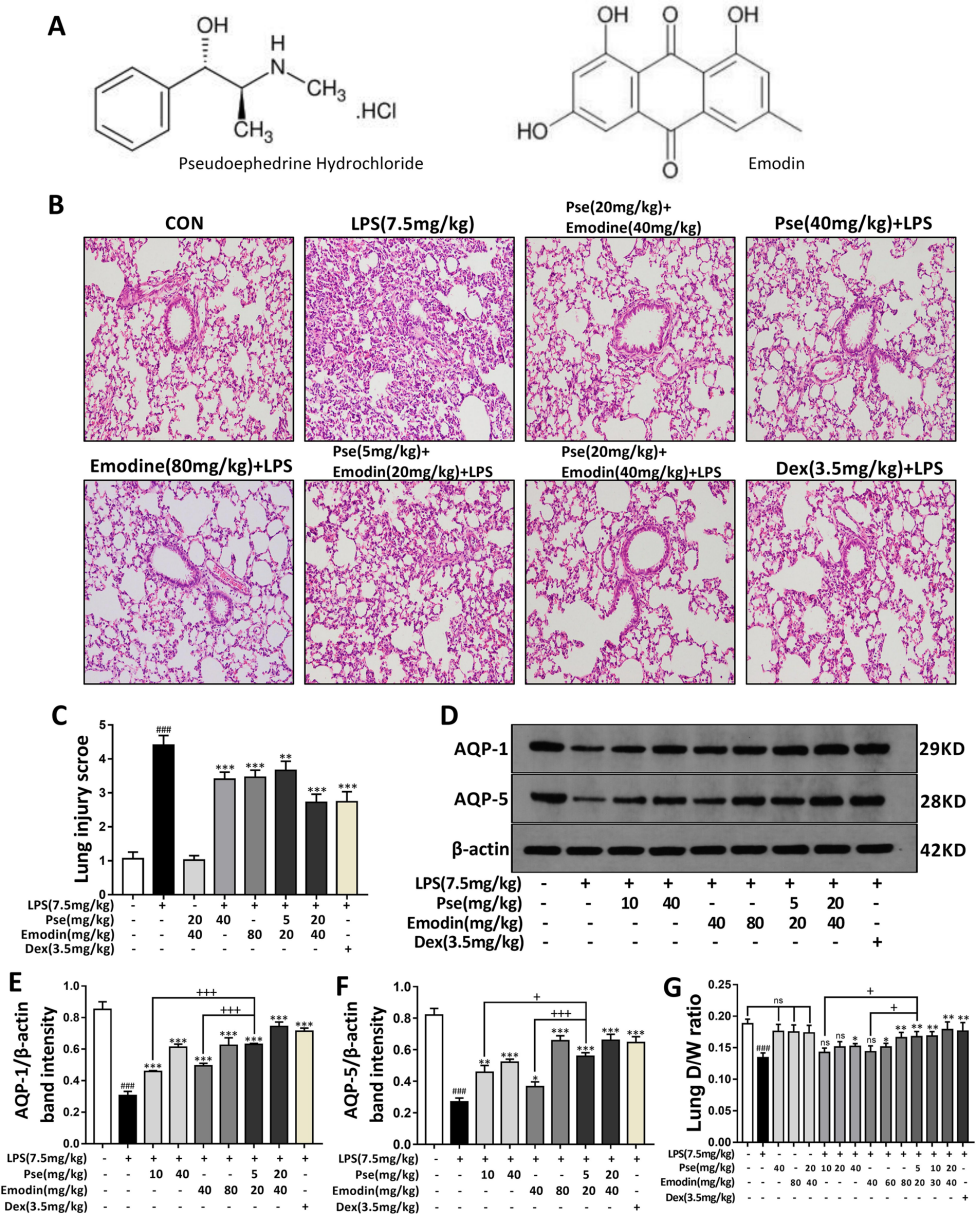
Effect of Pseudoephedrine + emodin on pathological changes of lung tissues (×200), expression of AQP‐1, AQP‐5 in LPS‐induced ALI rats. **A** The chemical structure of Pseudoephedrine and Emodin. **B** Stained with hematoxylin and eosin. **C** The lung injury score was determined following a five-point scale from 0 to 4 as follows: 0, l, 2, 3, and 4 represent no damage, mild damage, moderate damage, severe damage, and very severe damage, respectively. Representative histological sections of the lungs were stained with hematoxylin and eosin (H & E staining, magnification ×200). **D**–**F** Western blot analysis was performed to detect AQP‐1 and AQP‐5 protein expression. **K** The dry/wet weight ratio and lung weight/body weight ratio were used to reflect the pulmonary edema. All data are expressed as mean ± S.D. (n = 5). ^##^p < 0.01, ^###^p < 0.001 vs. control group. *p < 0.05, **p < 0.01, ***p < 0.001 vs. LPS alone group. ^+^p < 0.05, ^+++^p < 0.001 vs. combined treatment group (5 + 20 mg/kg)

The chemical structures of pseudoephedrine and emodin are shown in Fig. 1. After staining with hematoxylin–eosin, the lung tissues were evaluated by optical microscope, and an inflammatory score (0–5) was given according to the degree of histological change. The results showed that the lung interstitiums of the LPS group were thickened and had obvious neutrophil infiltrations and airway hemorrhages. Compared with the LPS group, the lung damage of the rats in the pseudoephedrine, emodin, and combined treatment groups was improved, and the lung damage in the high drug concentration group was lighter (Fig. 1C). The severity of pulmonary edema formation is judged by the D/W ratio and the protein expressions of AQP-1 and AQP-5. The results of the Western blot analysis showed that the protein expressions of AQP-1 and AQP-5 in the lung tissues of the LPS-induced ALI rats were inhibited. Furthermore, the expressions of AQP-1 and AQP-5 increased in the pseudoephedrine group (10, 40 mg/kg), the emodin group (40, 80 mg/kg), the combined treatment group (5 + 20, 20 + 40 mg/kg), and the Dex group (3.5 mg/kg). Interestingly, even when the doses were halved, the expressions of AQP-1 and AQP-5 in the combined treatment group (5 + 20 mg/kg) were significantly higher than those in the pseudoephedrine group (10 mg/kg) or the emodin group (40 mg/kg) (Fig. 1D–F). Lung tissue D/W ratios are used to intuitively reflect the extent of pulmonary edema. When pseudoephedrine or emodin were used alone or in combination therapy, the D/W ratio had an increasing trend as the drug concentrations increased, and the corresponding combined treatments had more obvious positive effects on pulmonary edema (Fig. 1G).

### Pseudoephedrine + emodin inhibits the overexpression of inflammatory factors and promotes secretion of immunosuppressive factors

**Fig. 2:**
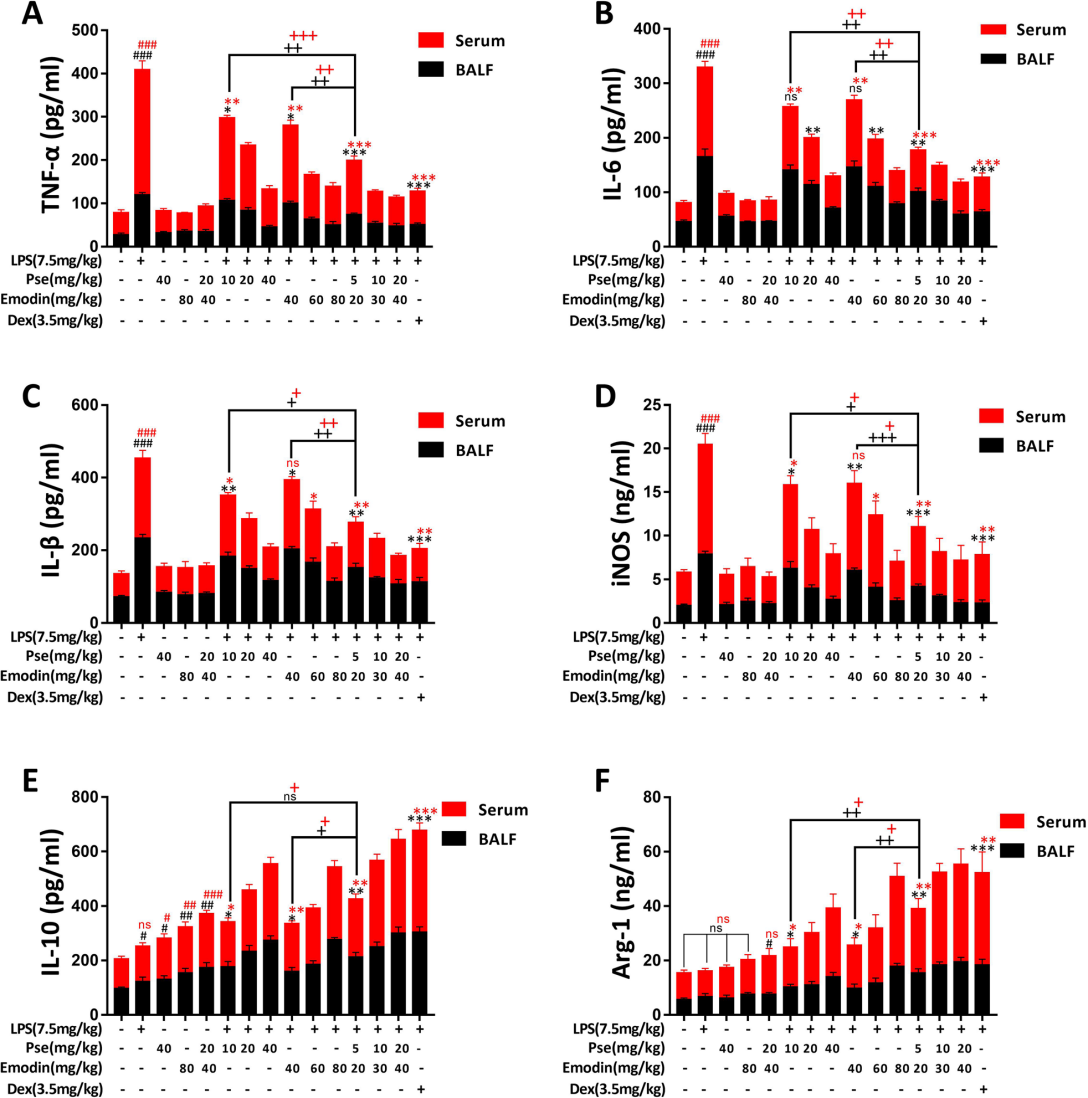
Effects of Pseudoephedrine + emodin on the expression of TNF-α, IL-6, IL-1β, iNOS, IL-10, Arg-1 in LPS‐induced ALI rats. The contents of TNF-α (**A**), IL-6 (**B**), IL-1β (**C**), iNOS (**D**), IL-10 (**E**), Arg-1 (**F**) in the serum and BALF were determined using ELISA. Data were expressed as mean ± S.D. (n = 3). ^#^p < 0.05, ^##^p < 0.01, ^###^p < 0.001 vs. control group. *p < 0.05, **p < 0.01, ***p < 0.001 vs. LPS alone group. ^+^p < 0.05, ^++^p < 0.01, ^+++^p < 0.001 vs. combined treatment group (5 + 20 mg/kg)

ELISA was used to measure the expressions of common inflammatory and anti-inflammatory factors in rat BALF and serum to evaluate the effects of different drug concentrations and single-drug or combination treatments on the inflammation levels of ALI rats. As shown in Fig. 2A–D, the levels of TNF-α, IL-6, IL-1β, and iNOS in the serum and BALF of rats in the LPS group were significantly increased. The levels of these inflammatory factors in the pseudoephedrine group (10, 40 mg/kg), the emodin group (40, 80 mg/kg), the combined treatment group (5 + 20, 20 + 40 mg/kg), and the Dex group (3.5 mg/kg) were significantly inhibited, and the inhibitory effect increased with greater drug concentrations. Moreover, our results showed that the combined treatments may have a greater inhibitory effect on inflammatory factors.

### Pseudoephedrine + emodin affects febrility in ALI rats

**Fig. 3:**
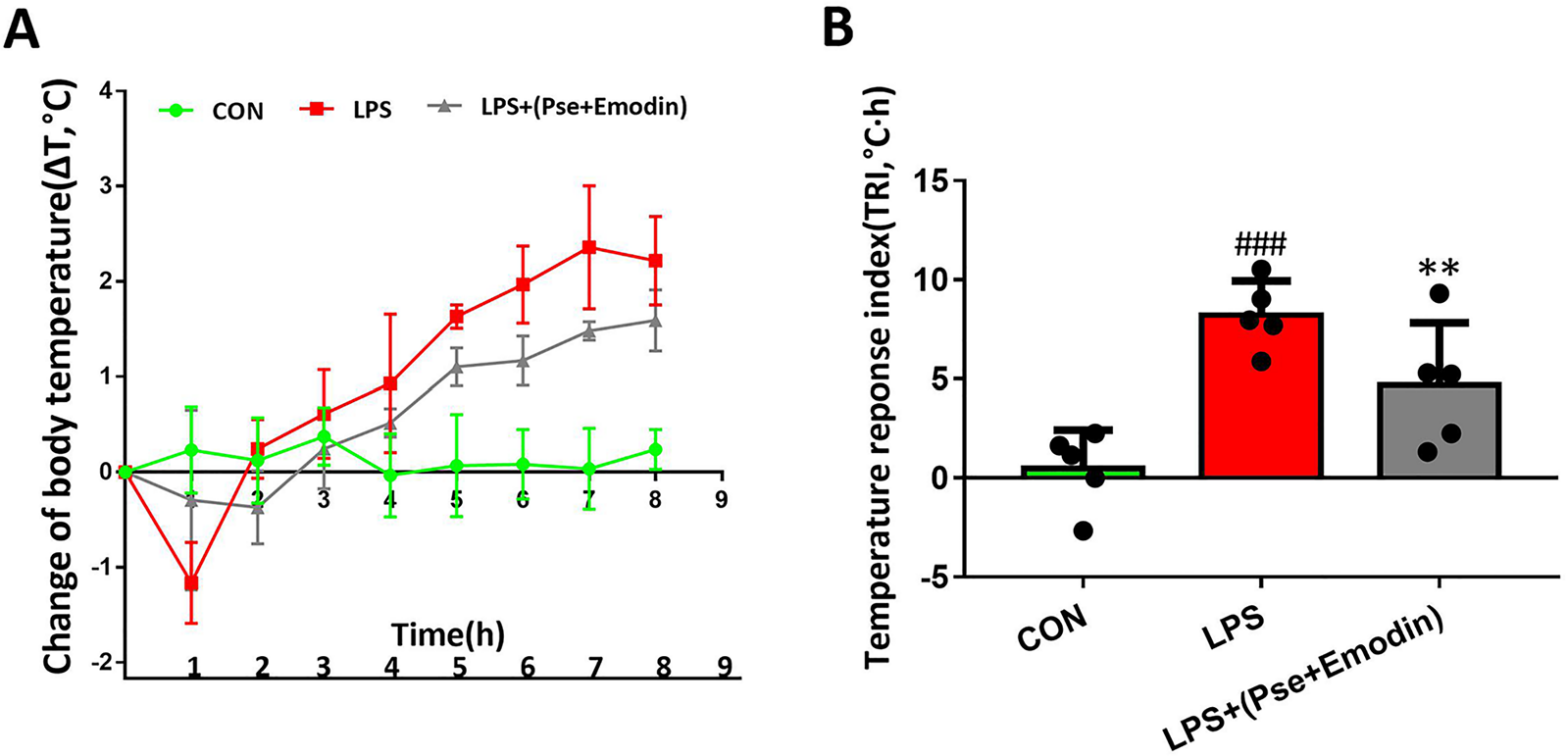
Effect of pseudoephedrine + emodin on febrility in LPS-induced ALI rats. **A**, **B** Changes in body temperatures and TRIs. All data are expressed as mean ± S.D. (n = 5). ^###^p < 0.001 vs. control group. **p < 0.01 vs. LPS group

In the control group, rat body temperatures remained within normal range throughout the experiment, with no significant changes occurring within 8 h of saline injection (Fig. 3A, B). In contrast, rats in the LPS group exhibited significant increase in body temperature and TRI, which began as an immediate decrease within 1 h after LPS injection, before significantly increasing between one to eight hours after injection. In the combined treatment group, the body temperatures and TRIs of the rats were significantly lower than those of the LPS group, which indicated the effectiveness of the pseudoephedrine + emodin treatment in alleviating febrility in LPS-induced ALI rats.

### VIP intervention affects pro-inflammatory and anti-inflammatory factor expression in LPS + IFN-γ, IL-4 + IL-13 induced alveolar macrophages

Macrophages play an important role in immune response. Considering the positive role of VIP in immune regulation. Therefore, we isolated and cultured alveolar macrophages from rats, used LPS + IFN-γ and IL-4 + IL-13 to induce macrophage polarization into M1 and M2 macrophages, respectively, and then studied the expression levels of pro- and anti-inflammatory cytokines after pretreatment with different concentrations of VIP.

**Fig. 4:**
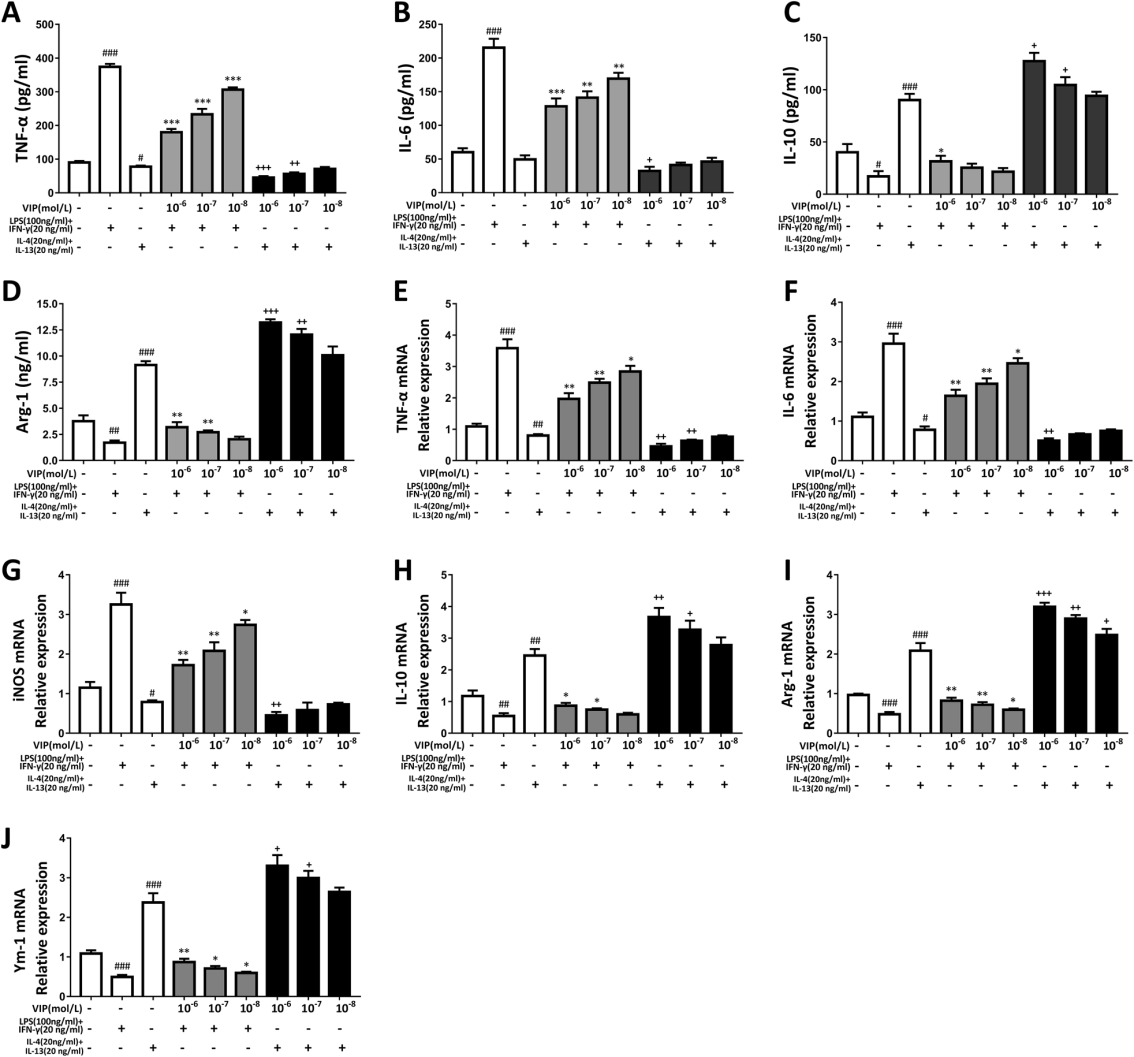
Effects of VIP on levels of inflammatory and anti-inflammatory cytokines in LPS + IFN-γ or IL-4 + IL-13 induced AMs cells. Macrophages were pre-treated with VIP(10^–6^,10^–7^, 10^–8^ mol/L) for 24 h, followed by LPS (100 ng/mL) + IFN-γ(20 ng/mL) or IL-4 (40 ng/mL) + IL-13(20 ng/mL) stimulation for 12 h. Cells were collected and the contents of TNF-α (**A**), IL-6 (**B**), IL-10 (**C**), Arg-1 (**D**) were determined using ELISA. (E-J) TNF-α, IL-6, iNOS, IL-10, Arg-1, Ym-1 mRNA expression was determined using Real-time PCR analysis. Data were expressed as mean ± S.D. (n = 3). ^#^p < 0.05, ^##^p < 0.01, ^###^p < 0.001 vs. control group. *p < 0.05, **p < 0.01, ***p < 0.001 vs. LPS alone group. +p < 0.05, ^+ +^p < 0.01, ^+++^p < 0.001 vs. IL-4 alone group

Figure 4 shows ELISA and real-time PCR results in measuring the expression levels of target cytokine proteins and mRNAs. In the LPS group, the expression of TNF-α, IL-6, and iNOS proteins and mRNA were significantly increased compared with the control group, while the expression of the anti-inflammatory IL-10, Arg-1, and Ym-1 proteins and mRNAs were decreased; macrophage polarization towards M1 was significant. In the IL-4-induced group, the levels of pro-inflammatory-related factors were only slightly increased compared with the control group. There were significant increases in the expression of IL-10, Arg-1, and Ym-1 proteins and mRNAs, and macrophage polarization towards M2 was significant. Increased VIP dose concentration resulted in a decrease in the expression of the pro-inflammatory TNF-α, IL-6, and iNOS proteins and mRNAs, as well as an overall increase in the expression of the anti-inflammatory IL-10, Arg-1, and Ym-1 proteins and mRNAs (Fig. 4).

These results indicate that VIP could effectively inhibit the release of pro-inflammatory factors and increase the expression of anti-inflammatory factors in macrophages.

### VIP intervention affects alveolar macrophage polarization

**Fig. 5:**
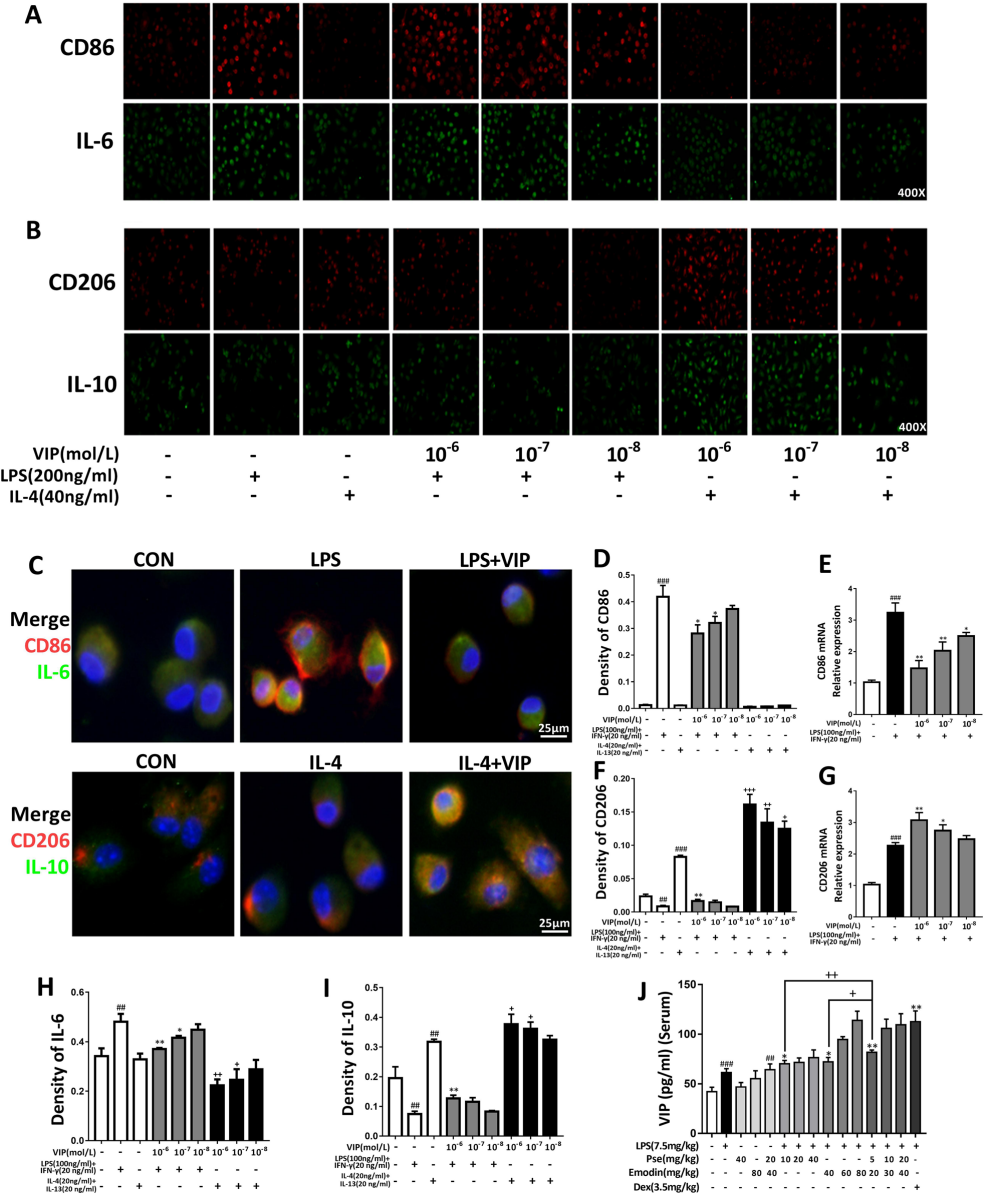
VIP inhibited (M1) macrophage and activated (M2) macrophage in LPS + IFN-γ‐induced or IL-4 + IL-13-induced AMs. Effects of Pseudoephedrine + emodin on the expression of VIP in LPS‐induced ALI rats. **A**–**D**, **F**, **H**, **I** (M1) macrophage Marker CD86, IL-6 and (M2) macrophage Marker CD206, IL-10 expression was determined using immunofluorescence. **E**, **G** CD86, CD206 mRNA expression was determined using Real-time PCR analysis. **J** The contents of VIP in the serum of ALI rats were determined using ELISA. Data are expressed as mean ± S.D. (n = 3). ^##^p < 0.01, ^###^p < 0.001 vs. control group. *p < 0.05, **p < 0.01 vs. LPS alone group. +p < 0.05, ^++^p < 0.01, ^+++^p < 0.001 vs. IL-4 alone group

CD86 and CD206 are known surface markers of M1 and M2 macrophages, respectively. In this study, the alveolar macrophages after VIP pretreatment were polarized by LPS + IFN-γ and IL-4 + IL-13, followed by immunofluorescence staining to label CD86, IL-6, and CD206, IL-10, respectively. The results are shown in Fig. 5A–I. In the alveolar macrophages that had been induced by LPS, high expressions of the macrophage surface marker CD86 could be observed, while in the VIP-pretreated cells, M1 polarization was significantly inhibited. Similarly, the expression of CD206 on the surface of macrophages increased after IL-4 induction, and M2 polarization increased after VIP pretreatment. The PCR results were consistent; compared with LPS or IL-4 induction, VIP pretreatment reduced the expression of CD86 mRNA and increased the expression of CD206 mRNA. These results showed that VIP, as an immunologically active neuropeptide, has the two-way regulatory effect of inhibiting the polarization of M1 macrophages and promoting the polarization of M2 macrophages. At the same time, in animal experiments, we found that both pseudoephedrine (10, 20, 40 mg/kg) and emodin (40, 60, 80 mg/kg) alone can increase the level of VIP in the serum of LPS-induced ALI rats. It is worth mentioning that we found that the combined treatment group (5 + 20, 10 + 30, 20 + 40 mg/kg) had higher serum VIP levels than the two separate treatment groups (Fig. 5J).

### Pseudoephedrine + emodin treatment induces VIP/cAMP/PKA signaling pathway and inhibits NF-κB signaling pathway

**Fig. 6:**
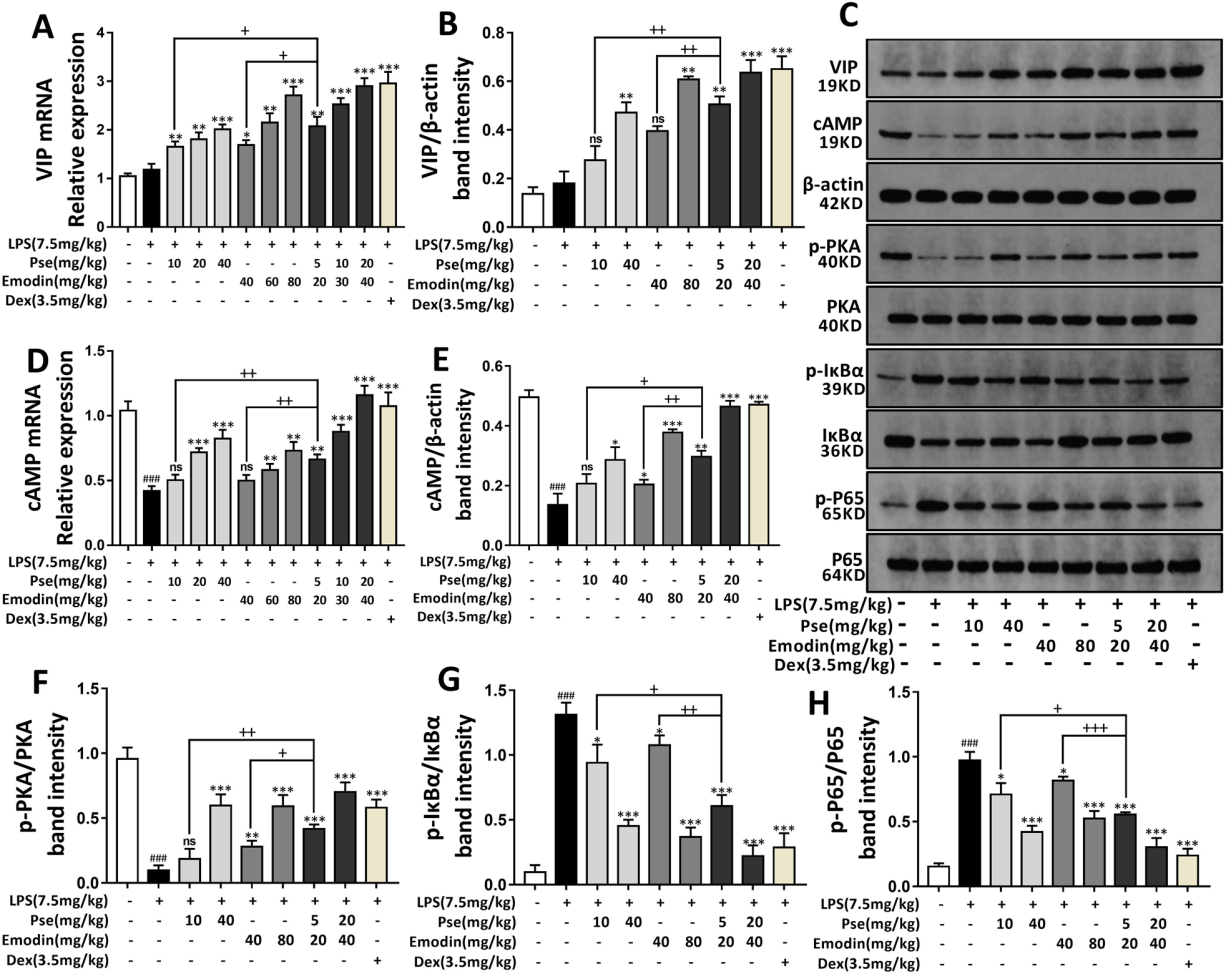
Pseudoephedrine + emodin up-regulated VIP/CAMP/PKA pathways and Inhibited NF-κB in LPS-induced acute lung injury in rats. **A**, **D** VIP, cAMP mRNA expression was determined using Real-time PCR analysis. **B**, **C**, **E**–**H** Western blot analysis was performed to detect VIP, cAMP, p-PKA, p-IκBα and p-P65 protein expression. All data are expressed as mean ± S.D. (n = 3). ^##^p < 0.01, ^###^p < 0.001 vs. control group. *p < 0.05, **p < 0.01, ***p < 0.001 vs. LPS alone group. ^+^p < 0.05, ^++^p < 0.01, ^+++^p < 0.001 vs. combined treatment group (5 + 20 mg/kg)

Previous experiments have confirmed that VIP can effectively promote the M2 polarization of alveolar macrophages to exert immunomodulatory function, and that the combined treatment of pseudoephedrine and emodin increases the level of VIP in the serum of ALI rats (Fig. 5J). To further study the protective mechanism of pseudoephedrine and emodin treatment on ALI rats, the mRNA levels of VIP and cAMP were detected by PCR, and the protein levels of VIP, cAMP, and p-PKA were analyzed by Western blot. As shown in Fig. 6A–F, LPS induced slight increases in the protein and mRNA levels of VIP and cAMP in the lung tissues of ALI rats, but significantly inhibited the level of p-PKA protein. After treatment, the protein and mRNA levels of VIP and cAMP in the pseudoephedrine group (10, 40 mg/kg), the emodin group (40, 80 mg/kg), the combined treatment group (5 + 20, 20 + 40 mg/kg) and the Dex group (3.5 mg/kg) were higher than those in the LPS group. Furthermore, the phosphorylation levels of PKA protein in these groups were also significantly enhanced compared with those in the LPS group. After comparison, the VIP/cAMP/PKA signal axis activation of the combined treatment group was more obvious than that of the single treatment group. The NF-κB signaling pathway is a typical inflammatory pathway induced and activated by LPS, and the VIP/cAMP/PKA signaling axis can effectively inhibit its activity. This study’s examination of the NF-κB signaling pathway protein expression of each group showed that a combined pseudoephedrine + emodin treatment could block the phosphorylation and degradation of IκBα in ALI induced by LPS and significantly inhibit the relative phosphorylation of P65 (Fig. 6G, H).

### Combined pseudoephedrine + emodin treatment inhibits the MAPK signaling pathway

**Fig. 7:**
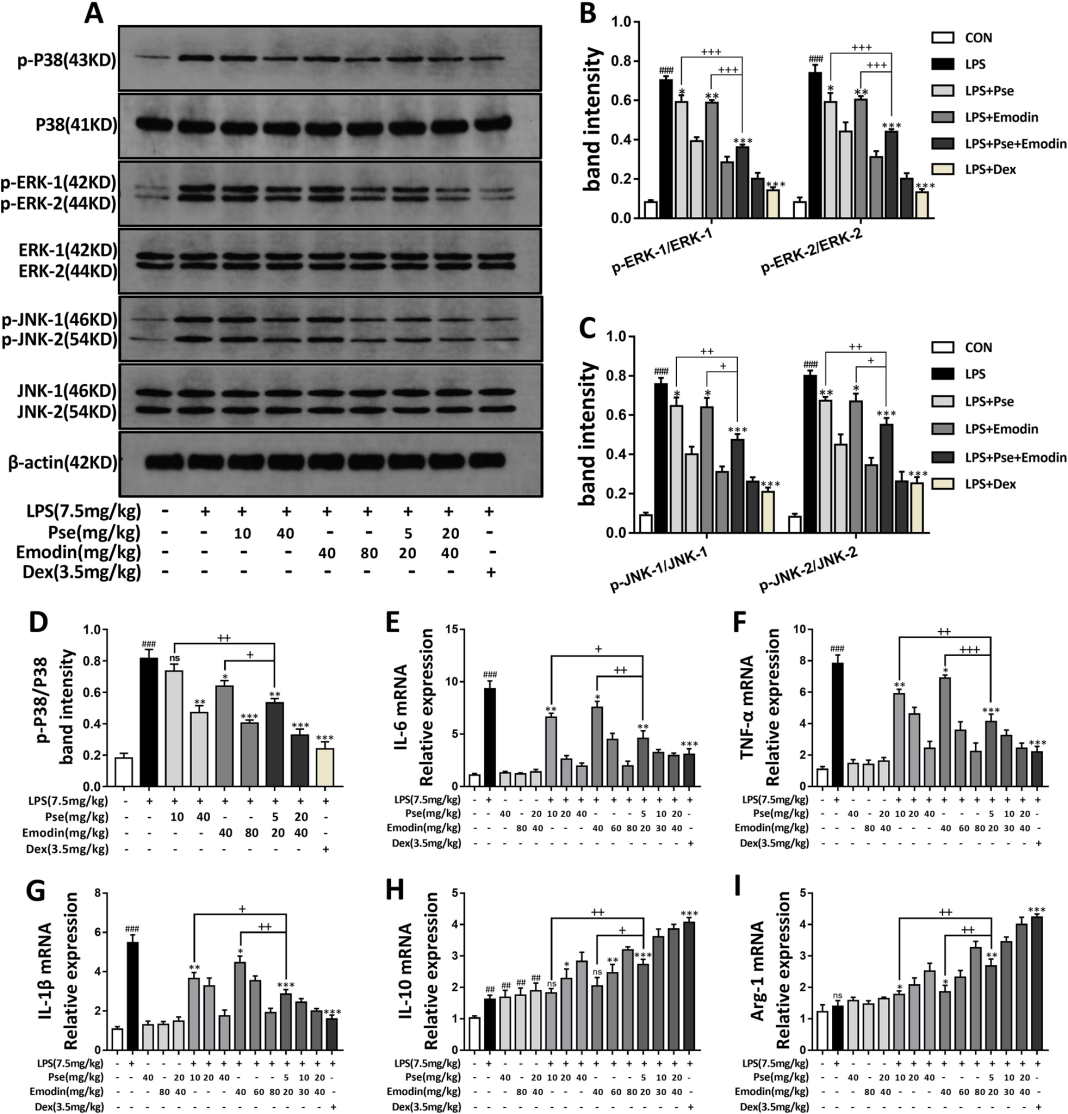
Pseudoephedrine + emodin Inhibited MAPK in LPS-induced acute lung injury in rats. **A**–**D** Western blot analysis was performed to detect p-P38, p-ERK1/2 and p-JNK1/2 protein expression. **E**–**I** TNF-α, IL-6, IL-1β, IL-10, Arg-1 mRNA expression was determined using Real-time PCR analysis. All data are expressed as mean ± S.D. (n = 3). ^##^p < 0.01, ^###^p < 0.001 vs. control group. *p < 0.05, **p < 0.01, ***p < 0.001 vs. LPS alone group. ^+^p < 0.05, ^++^p < 0.01, ^+++^p < 0.001 vs. Combined treatment group (5 + 20 mg/kg)

The MAPK pathway is composed of the P38, ERK, and JNK signaling pathways, which can be activated by pro-inflammatory factors and play key roles in the development of inflammation. To explore the anti-inflammatory effect of pseudoephedrine and emodin combined treatment on the MAPK signaling pathway in the lung tissue of LPS-induced ALI rats, we used Western blots to detect the phosphorylation levels of the P38, ERK1/2, and JNK1/2 proteins in the tissue. The results showed that pseudoephedrine + emodin pretreatment significantly inhibited the relative phosphorylation levels of P38, ERK1/2, and JNK1/2 induced by LPS. There were similar treatment effects in the positive treatment group, and it was also found that the combined treatment had a better inhibitory effect on the MAPK pathway than the single treatment (Fig. 7A–D). With PCR, we detected the mRNA levels of pro-inflammatory factors (TNF-α, IL-6, and IL-1β) and anti-inflammatory factors (IL-10 and Arg-1) in the lung tissues. The results showed the same trends as seen in their levels in BALF and serum, and the combined treatment showed better anti-inflammatory abilities than the single treatment group (Fig. 7E–I).

### Pseudoephedrine + emodin treatment inhibits M1 polarization and promotes M2 polarization in alveolar macrophages

**Fig. 8:**
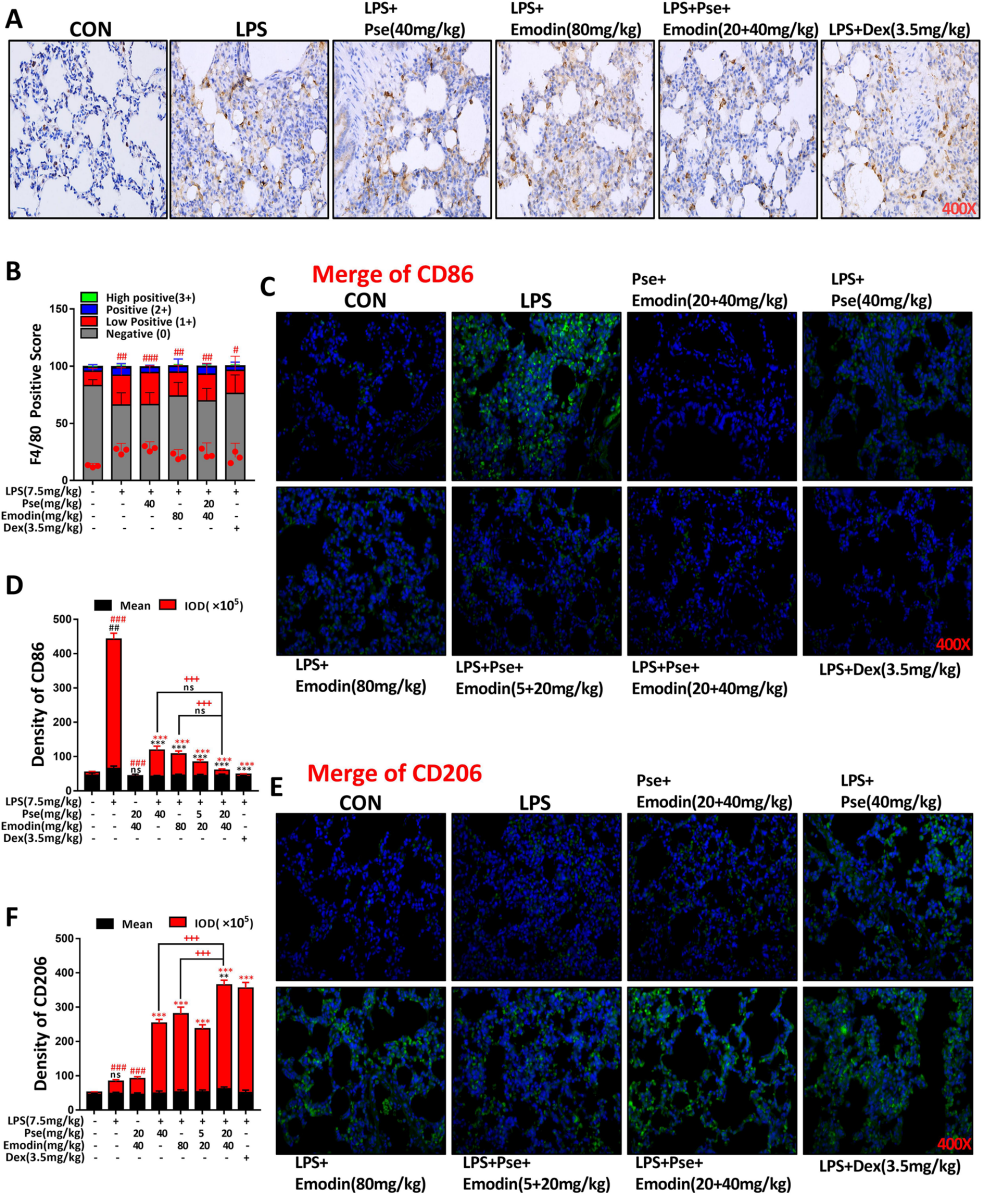
Pseudoephedrine + emodin inhibited (M1) macrophage and activated (M2) macrophage in LPS‐induced rats lung tissue. **A** F4/80 expression was determined using immunohistochemistry (n = 3). **B** F4/80 positive score was determined using ImageJ: negative (0), low positive (1+), positive (2+), high positive (3+). **C**–**F** CD86, CD206 expression was determined using immunofluorescence (n = 5). Data are expressed as mean ± S.D. ^##^p < 0.01, ^###^p < 0.001 vs. control group. **p < 0.01, ***p < 0.001 vs. LPS alone group. ^+++^p < 0.001 vs. combined treatment group (20 + 40 mg/kg)

F4/80 is a macrophage surface marker that is commonly used to reflect macrophage activation status and recruitment levels. To determine the effects of pseudoephedrine + emodin on the polarization of macrophages in LPS-induced rat lung tissues, we performed immunohistochemical analysis of F4/80 on rat lung tissue sections and performed immunofluorescence analysis of CD86, CD206. Results indicate that the macrophage surface marker F4/80 was highly expressed in both the LPS group and each treatment group compared with the control group. It is suggested that macrophages of these group were active except the control group (Fig. 8A, B). However, differently, from the immunofluorescence results, we can see that LPS induced group macrophages were dominated by M1 polarization, forming a proinflammatory environment in lung tissue; However, M2 polarization was predominant in the group treated with monotherapy, combination therapy, or DEX, where it was stronger in the combination versus DEX group, creating a favorable anti-inflammatory environment (Fig. 8C–F).

## Discussion

Inflammation is an important part of the immune response that protects the body from pathogens, but excessive inflammation can cause tissue and organ damage [28]. More and more evidence shows that a variety of pathological changes in lung tissue occur at the onset of ALI. These changes can be increased capillary permeability, extensive neutrophil infiltration, release of inflammatory mediators, and pulmonary edema, among others [29]. The homeostasis of water is the basis for cell survival, and the transport of water across the cell membrane is controlled by aquaporin (AQP. AQP-1 and AQP-5 have similar structural characteristics: AQP-1 mainly regulates pulmonary interstitial edema caused by increased pulmonary circulation pressure; AQP-5 is closely related to the transmembrane transport of water molecules in alveolar epithelial cells and plays a key role in the secretion of isotonic fluid [30]. The results of this study showed that a combined pseudoephedrine + emodin treatment could effectively restore the protein expression of AQP-1 and AQP-5 in the lung tissue of LPS-induced ALI rats. At the same time, the combined treatment increased the D/W ratio of lung tissue, which improved the reduction of pulmonary edema. In addition, the pathological observation of HE stains also found that the infiltration of neutrophils in the lung tissue of ALI rats treated was significantly reduced with the combination treatment.

Inflammatory factors such as TNF-α, IL-6, IL-1β, and iNOS are closely related to the development of acute and chronic inflammatory diseases [31, 32]. ALI disease is often accompanied by a large release of these inflammatory factors, which damage the lung tissue. The uncontrolled accumulation of inflammatory factors can also cause sepsis and multiple organ dysfunction syndrome [6, 7]. In this study, a rat model of septic ALI induced by intraperitoneal injection of LPS was used, and the BALF and serum levels of inflammatory and anti-inflammatory factors in each group of rats were detected to investigate whether the intervention of pseudoephedrine and emodin has a therapeutic effect on sepsis and lung injury. Our findings showed that combined pseudoephedrine + emodin treatment could significantly inhibit the secretion of inflammatory factors (TNF-α, IL-6, IL-1β, and iNOS) and promote the secretion of anti-inflammatory factors (IL-10 and Arg-1) in BALF and serum. In addition, fever is a classic clinical manifestation of inflammation. The body temperature of the ALI rats increased significantly within 2–8 h after LPS induction, and the fever symptoms of the rats were significantly relieved after the combined treatment. In short, this evidence indicates that the combination of pseudoephedrine and emodin alleviates septic lung injury in rats.

In the inflammatory response, M2 macrophages are often the key to suppressing excessive inflammatory responses [33]. As an anti-inflammatory neuropeptide, VIP plays an important role in immune regulation [34]. VIP is a negative regulator of inflammation, promoting the generation of an anti-inflammatory microenvironment by regulating the function and distribution of monocytes, macrophages, and regulatory T cells [35]. In the cell experiments, we verified that VIP could effectively inhibit the mRNA expression of pro-inflammatory factors (TNF-α, IL-6, TNF-α, and induced iNOS) in M1 and M2 macrophages and increase the protein and mRNA expressions of the anti-inflammatory factor (IL-10, Arg-1, and Ym-1). The subsequent immunofluorescence results further confirmed the regulation effect of VIP on the polarization of M2 macrophages.

The mechanism by which VIP inhibits M1 polarization and promotes M2 polarization may be related to the inhibition of the NF-κB signaling pathway. Various inflammatory mediators mediated by the NF-κB signaling pathway play a vital role in the pathogenesis of ALI. Furthermore, the NF-κB signaling pathway is the key to the activation of pro-inflammatory factors [36–38]. It has been reported that VIP can bind to the VPAC1 receptor of polarized M1 macrophages in vitro to increase the expression levels of cAMP and p-PKA while simultaneously inhibiting IκBα phosphorylation to down-regulate the transcriptional activity of the NF-κB (p65) signaling pathway [39]. In animal experiments, we found that LPS induced an increase in VIP concentrations in the serum of ALI rats. It is speculated that this increase is the manifestation of the rat's autoimmune regulation. At the same time, the serum VIP concentrations of the ALI rats that received the combination treatment had significantly increased compared to those of the untreated rats. Next, we confirmed that the combination of pseudoephedrine and emodin could activate the expression of the VIP/cAMP/PKA signal axis in the lung tissue of the ALI rats, significantly inhibiting IκBα phosphorylation and the dissociation of p65 and the phosphorylation of p65 in the nucleus, thereby blocking the transcription and expression of inflammatory mediators and immune-related genes.

To fully evaluate the protective effects and advantages of the combined pseudoephedrine + emodin treatment on ALI, we focused on the MAPK signaling pathway that responds to LPS-induced lung inflammation. Previous studies have shown that the MAPK signaling pathway is closely related to acute and chronic inflammatory diseases and can be activated by stimuli such as LPS and TNF-α. After activation of the MAPK signaling pathway, phosphorylated ERK, p38, and JNK transfer to the nucleus, induce the expression of target genes of related inflammatory factors, and promote an inflammatory response [40, 41]. This study found that the combined pseudoephedrine + emodin treatment can significantly inhibit the phosphorylation of MAPK P38, ERK1/2, and JKN1/2. The experimental results also showed that the combined treatment has a stronger inhibitory effect on the MAPK signaling pathway than a single treatment of pseudoephedrine or emodin alone.

**Fig. 9:**
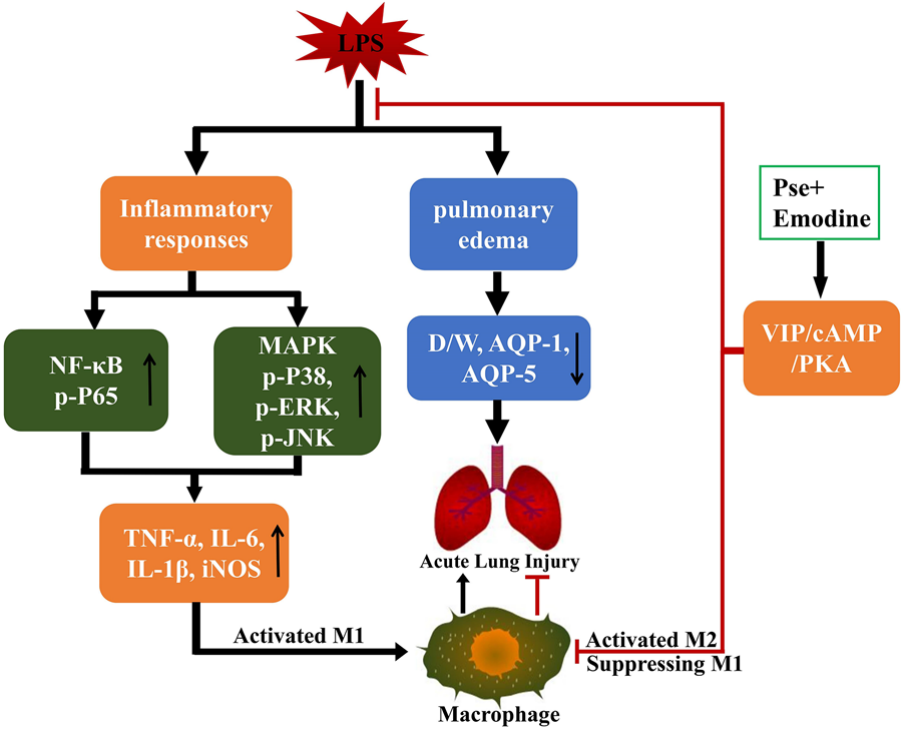
Scheme summarizing the protective effects of pseudoephedrine and emodin on LPS-induced acute lung injury via activation of the VIP/cAMP/PKA axis

Previous cell experiments found that VIP treatment could inhibit the polarization of M1 macrophages and promote the polarization of M2 macrophages in rat alveoli. In our animal experiments, we found that the combination of pseudoephedrine and emodin could increase the content of VIP in the serum of LPS rats and activate the VIP/cAMP/PKA signal axis. Interestingly, the results of immunohistochemistry and fluorescence showed that the active macrophages were polarized from M1 to M2 in the lung tissues of the ALI rats that received the combined treatment, and the M2 polarization trend was more obvious in the combined treatment group than in the single treatment group. This may be related to the inhibition of core inflammatory pathways, such as NF-κB and MAPK, by the immune regulation signal axis dominated by VIP/cAMP/PKA (Fig. 9).

### Conclusions

In conclusion, our findings prove that the combined treatment of pseudoephedrine and emodin could effectively reduce pulmonary edema and inflammatory damage in ALI. The pulmonary edema and inflammatory damage was largely related to the activation of the VIP/cAMP/PKA signal axis; therefore, the combined treatment would restore the expression of AQP-1 and AQP-5 proteins, inhibit the NF-κB and MAPK signaling pathways, inhibit the polarization of M1 macrophages, and promote the polarization of M2 macrophages. Our study comprehensively evaluated the effect of combined treatment on these related factors, and the results provide useful evidence for the combination of pseudoephedrine and emodin in the treatment of ALI.

## Data Availability

The datasets used in the current study are available from the corresponding author on reasonable request.

## Abbreviations

ALI: Acute lung injury
LPS: Lipopolysaccharide
IL-6: Interleukin-6
TNF-α: Tumor necrosis factor-α
G-CSF: Granulocyte colony-stimulating factors
VIP: Vasoactive intestinal peptide
cAMP: Cyclic adenosine monophosphate
ACs: Activate adenylate cyclases
PKA: Protein kinase
ARIs: Acute respiratory infections
BALF: Bronchoalveolar lavage fluid
H&E: Haematoxylin and eosin
ELISA: Enzyme-linked immunosorbent assay
WB: Western blotting
